# Enhanced Solar Photocatalytic Reduction of Cr(VI) Using a (ZnO/CuO) Nanocomposite Grafted onto a Polyester Membrane for Wastewater Treatment

**DOI:** 10.3390/polym13224047

**Published:** 2021-11-22

**Authors:** Ambreen Ashar, Ijaz Ahmad Bhatti, Asim Jilani, Muhammad Mohsin, Sadia Rasul, Javed Iqbal, Muhammad Bilal Shakoor, Abdullah G. Al-Sehemi, S. Wageh, Ahmed A. Al-Ghamdi

**Affiliations:** 1 Radiation Chemistry Lab, Department of Chemistry, University of Agriculture Faisalabad (UAF), Faisalabad 38000, Pakistan; ambreenashar2013@gmail.com (A.A.); ijazchem@yahoo.com (I.A.B.); sadiaakashif11@yahoo.com (S.R.); 2 Center of Nanotechnology, King Abdulaziz University, Jeddah 21589, Saudi Arabia; iqbaljavedch@gmail.com; 3 College of Earth & Environmental Sciences, University of the Punjab, Lahore 54590, Pakistan; bilalshakoor88@gmail.com; 4 Research Center for Advanced Materials Science (RCAMS), Department of Chemistry, Faculty of Science, King Khalid University, P.O. Box 9004, Abha 61413, Saudi Arabia; agmasq@gmail.com; 5 Department of Physics, Faculty of Science, King Abdulaziz University, Jeddah 21589, Saudi Arabia; wswelm@kau.edu.sa (S.W.); agamdi@kau.edu.sa (A.A.A.-G.); 6 Physics and Engineering Mathematics Department, Faculty of Electronic Engineering, Menoufia University, Menouf 32952, Egypt

**Keywords:** chromium (VI) reduction, ZnO/CuO/PF photocatalytic membrane reactor, response surface methodology

## Abstract

Among chemical water pollutants, Cr(VI) is a highly toxic heavy metal; solar photocatalysis is a cost-effective method to reduce Cr(VI) to innocuous Cr(III). In this research work, an efficient and economically feasible ZnO/CuO nanocomposite was grafted onto the polyester fabric ZnO/CuO/PF through the SILAR method. Characterization by SEM, EDX, XRD, and DRS confirmed the successful grafting of highly crystalline, solar active nanoflakes of ZnO/CuO nanocomposite onto the polyester fabric. The grafting of the ZnO/CuO nanocomposite was confirmed by FTIR analysis of the ZnO/CuO/PF membrane. A solar photocatalytic reduction reaction of Cr(VI) was carried out by ZnO/CuO/PF under natural sunlight (solar flux 5–6 kW h/m^2^). The response surface methodology was employed to determine the interactive effect of three reaction variables: initial concentration of Cr(VI), pH, and solar irradiation time. According to UV/Vis spectrophotometry, 97% of chromium was removed from wastewater in acidic conditions after four hours of sunlight irradiation. ZnO/CuO/PF demonstrated reusability for 11 batches of wastewater under natural sunlight. Evaluation of Cr(VI) reduction was also executed by complexation of Cr(VI) and Cr(III) with 1, 5-diphenylcarbazide. The total percentage removal of Cr after solar photocatalysis was carried out by AAS of the wastewater sample. The ZnO/CuO/PF enhanced the reduction of Cr(VI) metal from wastewater remarkably.

## 1. Introduction

Water is a basic requirement for all life forms on earth, but availability of fresh water is limited due to the poor management of industrial wastewater that is directly discharged into waste streams without any treatment [1]. This contaminated water can be reused for cultivation and drinking purposes after passing through effective treatment in order to overcome this great issue [2]. In industrial wastewater, the major environmental pollutants that adversely affect the ecological food chain are heavy metals [3]. A number of heavy metals such as chromium (Cr), nickel (Ni), lead (Pb), cadmium (Cd), mercury (Hg), zinc (Zn), silver (Ag), and copper (Cu) are present in wastewater, making it poisonous for all life on land [4].

Cr(VI) is highly toxic heavy metal because it does not require a specific membrane carrier; it can penetrate through the cell membrane [5], which ultimately causes the oxidation of biological molecules. Cr(VI) can affect plant growth as it can accumulate in the plant body through its roots, reducing the process of photosynthesis [6]. Moreover, Cr(VI) highly affects the enzymatic and biological plant systems, such as in barley, wheat, maize, and vegetables and in cauliflower, it causes chlorosis and necrosis [7]. It can disturb the ecological food chain, as eating plants polluted plants chromium can cause diarrhea, nausea, lung tumors, and headaches in human beings; thus, wastewater treatment for chromium removal is essential for human and plant growth [8]. Some processes are generally used for the treatment of wastewater, such as membrane filtration, biodegradation, ion exchange, electrolysis, sorption, and coagulation [9,10]. However, these methods consume a high amount of energy, have a high sensitivity and high cost, and produce sludge during operation [11].

Solar photocatalysis is an advanced oxidation process that is broadly used for elimination of micropollutants from wastewater as it is highly cost-effective, easy to execute, and provides better removal efficiency for pollutants [12]. Currently, wastewater is widely treated by photocatalysis using a number of semiconductor nanomaterials, but their working efficiency can be enhanced by fabricating their composites. ZnO/CuO nanocomposite is one of the best nano-photocatalysts due to its low cost, environmentally friendly nature, enhanced photocatalytic activity, and higher chemical reactivity. Furthermore, ZnO is an n-type semiconductor having a wide band gap (3.37 eV) [13,14,15] and CuO is a p-type semiconductor having a narrow band gap (1.7 eV) [16]. On combining p-type CuO with n-type ZnO, the nanocomposite improves the photocatalytic activity in the visible region of the solar spectrum by enhancing electron–hole pair generation [17,18,19].

In heterogeneous photocatalysis, a photocatalyst is used in suspension as a slurry reactor, but it is not suitable for separation, reuse, and regaining of the photocatalyst for the treatment of the next batch of polluted water [14,20,21]. This can be overcome by immobilizing the photocatalyst onto a substrate such as glass, ceramics, or fabric-based membranes. Among the different substrates, membrane technology is the most widely used; so far, the reported membrane materials include polyamide, polyurethane, polyethylene, and polyacrylonitrile [22]. Polyester-based membranes are a good choice in membrane technology due to having the characteristics of high strength, high durability, quick drying, strong resistance against chemicals, no shrinking, no stretching, being wrinkle- free, and a hydrophobic nature [23]. Therefore, we used a polyester membrane as substrate for grafting a thin film of ZnO/CuO nanocomposite to form a photocatalytic membrane for Cr(VI) removal from wastewater.

A number of methods are widely used for thin film growth on substrates, such as sol-gel coating, pulsed laser deposition (PLD), electrochemical bath deposition, and chemical vapor deposition (CVD), but these methods all need complicated instruments and high temperature [24]. The successive ionic layer adsorption and reaction (SILAR) method has proven to be the best method for thin film growth as it is the most economical, the simplest, less time consuming, and can be easily executed at room temperature for grafting a photocatalyst on a large area [25].

On considering all the drawbacks of the previously reported methods for wastewater treatment and the immobilization of nanostructures over diverse kinds of substrates, in this research work, a thin film of ZnO/CuO nanocomposite has been grafted on polyester fabric (PE) by the successive ionic layer adsorption and reaction (SILAR) method and the fabricated material was characterized by EDX, SEM, and XRD. Three operational parameters, i.e., irradiation time, initial concentration of Cr(VI), and pH, were optimized by response surface methodology in order to achieve the maximum percentage reduction of Cr(VI). The fabricated photocatalytic membrane reactor (PMR) was further used for the photocatalytic reduction of Cr(VI) in real wastewater. The PMR designed is novel and reusable for a number of batches of wastewater without producing any secondary sludge. Reduction of Cr(VI) to Cr(III) in order to treat industrial wastewater is much more ecofriendly than adsorbing Cr(VI) on different adsorbents.

## 2. Experimental

### 2.1. Chemicals Required

Zinc acetate dehydrate (Zn(CH_3_COO)_2_·2H_2_O), copper acetate (Cu(CH_3_COO)_2·_2H_2_O), and potassium dichromate (K_2_Cr_2_O_7_) were purchased from Daejung. Sodium hydroxide (NaOH), sodium carbonate (Na_2_CO_3_), potassium permanganate (KMnO_4_), sulfuric acid (H_2_SO_4_), 1,5-diphenylcarbazide (C_13_H_14_N_4_O), and hydrogen peroxide (H_2_O_2_ 36%) were purchased from Merck. The untreated polyester fabric was procured from National Textile University, Faisalabad, Pakistan, and wastewater containing Cr(VI) ions was collected from the Rajawala drain near the University of Agriculture, Faisalabad, Pakistan.

### 2.2. Mercerization of Polyester Fabric

The procured untreated polyester fabric was washed with distilled water and ethanol to remove any impurities. Binding forces of polyester fabric were enhanced by rendering it hydrophilic with the surface deposition of hydroxyl groups (–OH), and a chemical treatment method named mercerization was employed. A one-meter square piece of polyester fabric (PF)was boiled in 40 g/L of NaOH with continuous stirring for 30 min and temperature was maintained at 60 °C. After cooling the solution, the polyester fabric was removed and the excessive NaOH was washed out with distilled water. Untreated polyester fabric was functionalized so that the nano-photocatalyst may graft evenly and densely onto its surface [26].

### 2.3. Fabrication of ZnO/CuO Nanocomposite on Polyester Fabric (ZnO/CuO/PF Membrane)

For the grafting of ZnO/CuO nanocomposite on the polyester fabric (by the SILAR method), during the first step a cationic complex of copper sodium zincate was formed by adding 0.05 M of Zn(CH_3_COO)_2_·2H_2_O, 0.05 M of Cu(CH_3_COO)_2_·2H_2_O, and 0.2 M of NaOH in 1:10 ratio. One piece (10 × 10 inch) of polyester fabric (PF) was firstly dipped in the cationic solution for 30 s then dipped in the anionic solution for 30 s to graft ZnO/CuO nanocomposite onto the PF membrane. This cycle was repeated 30 times to have a better growth of ZnO/CuO nanocomposite on the PF membrane.

**Figure 1:**
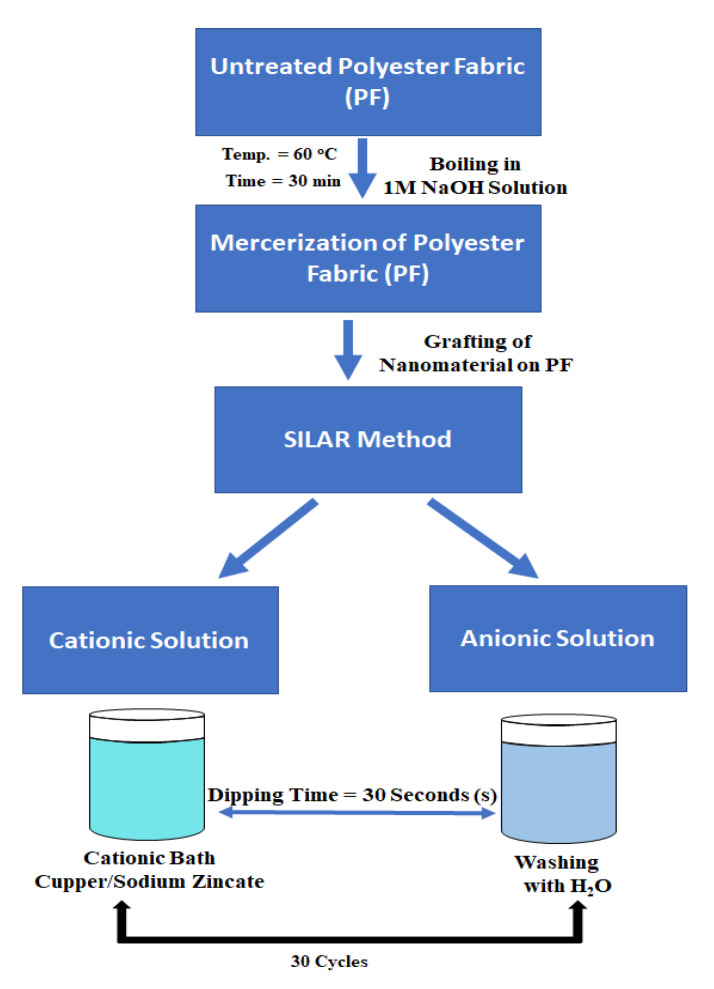
Schematic diagram of pathway followed for grafting of (ZnO/CuO) nanocomposite onto the surface of polyester.

In the second step, functionalized polyester fabric was cut into pieces of 10 cm^2^ and each piece was sequentially dipped in the above prepared cationic solution, in distilled water as the anionic solution for 30 s, then air-dried and the cycle was repeated 30 times. The surface grafted (–OH) groups react with the cationic solution in order to form ZnO/CuO nanocomposite (Figure 1) [27]. Finally, unreacted ions were removed from the ZnO/CuO coated polyester fabric by washing it with distilled water. After air drying all the fabricated PMRs, each side of them was exposed to UV light (intensity 44 W) for 30 min to bind the nanocomposite firmly onto the surface of the functionalized polyester fabric. The photocatalyst load on PMR was measured to be 58 ± 2 μg/cm^3^ by comparing the weight of the untreated polyester fabric and the PF loaded with ZnO/CuO nanocomposite.

### 2.4. Characterization of Fabricated ZnO/CuO/PF Membrane

ZnO/CuO nanocomposite was scraped from the surface of the functionalized polyester fabric and characterized to analyze its crystallinity, purity, and crystallite size by an X-ray diffractometer (Jeol JDX-3532, UK diffractometer) using CuKα irradiation (λ = 1.54 Å). The morphology, surface texture, and elemental analysis of ZnO/CuO nanocomposite was examined by using a scanning electron microscope (Quanta 2500, FEG (USA) and energy dispersive X-ray analysis (Oxford instruments, Abingdon, UK). The optical properties of the nanocomposite were determined by DRS (Perkin Elmer Lambda 1050, Buckinghamshire, UK). The FTIR analysis of the untreated polyester fabric and solar photocatalytic membrane loaded with ZnO/CuO nanocomposite was done by an IFS 125HR FTIR spectrometer (Bruker, Yokohama, Japan). For the assessment of thermal stability, TGA of ZnO/CuO nanocomposite was carried out by using a PerkinElmer Thermal Analyzer.

### 2.5. Evaluation of Concentration of Cr (VI) and Cr (III) by Complexation

The effluent containing hexavalent chromium formed a complex with 1, 5-diphenylcarbazide used as a complexing agent. First, 0.5 g of 1, 5-DPC in 100 mL of acetone was dissolved completely and then diluted with distilled water. Samples of Cr(VI) standard solution were prepared in concentrations from 10 to 50 ppm. The extraction reagents, NaOH (2%) and Na_2_CO_3_ (3%), were prepared in distilled water and dissolved in 0.1% potassium permanganate solution until a pink color was obtained. After mixing these reagents with Cr(VI) standards, 4 mL of sulfuric acid was added to obtain a pH of 2 (solution A). Then, 1, 5-DPC solution (2 mL) was mixed with solution A and a red-violet color of the Cr(VI) complex formed rapidly under the acidic conditions [28,29,30,31]. The concentration of Cr(III) was tested; (Cr(H_2_O)_6_)^3+^ was produced upon the addition of a small quantity of Na_2_CO_3_ in Cr(III), forming grey-green precipitates of tri-aqua, trihydroxy, and chromium (III) complex. The precipitates were filtered to obtain chromium-free water [32]. The total percentage removal of chromium after complexation was determined by ICP-MS (Agilent 7700×, Santa Clara, CA, USA).

#### Photocatalytic Reduction of Cr(VI) in Wastewater

Photocatalytic reduction was performed for standard solutions of Cr(VI) through a series of experiments in which pH was adjusted through acidic and basic solutions and the irradiation time of natural sunlight was varied using a ZnO/CuO/PE-based PMR. The PMRs of 5 × 5 cm^3^ size were cut and immersed in glass containers of 10 × 10 × 4 cm^3^ with a working volume of 50 mL of standard solutions of Cr(VI) and exposed to natural direct sunlight during 11 a.m.–3 p.m. in the month of May. The average temperature was about 35–40 °C and solar flux was 5–6 kW h/m^2^. The extent of reduction of Cr(VI) from the treated samples was measured by using a UV/Vis spectrophotometer, while percentage reduction was measured in terms of the absorbance of Cr(VI) before and after photocatalytic reduction using Equation (1) [31]. The equipment used for the measurement of absorption was a UV/Vis spectroscope (CE Cecil 7200, Isernhagen, Germany).

(1) $$\;\;\;\;\;\;\;\;\;\;\;\;\;\;\;\;\;\;\;Reduction\left(\%\right)=\frac{A_{o}-A_{f}}{A_{o}}\times 100$$

where $A_{0}$ is the initial absorbance and $A_{f}\;$ is the final absorbance. Calculated values were further used for the application of RSM to determine the results and relationship between independent variables.

### 2.6. Optimization of Operational Parameters by Response Surface Methodology

The variable operational parameters, i.e., pH (5–9), initial Cr(VI) concentration (10–50 ppm), and irradiation time (2–6 h), were statistically optimized through response surface methodology (RSM), which is a statistical and mathematical tool for optimization of reaction parameters for achieving the maximum reduction of Cr(VI). The basic equation for the selected quadratic model that represents the relationship between variables and their interactive effect on the percentage reduction of Cr(VI) is given in Equation (2).

Y = β* + β_1_X_1_ + β_2_X_2_ + β_3_X_3_ + β_12_X_1_X_2_ + β_13_X_1_X_3_ + β_23_X_2_X_3_ + β_1_^2^X_1_^2^ + β_2_X_2_^2^ + β_3_X_3_^2^ (2)

The model suggested by the central composite design of RSM was applied on the abovementioned values of variables and 20 runs were conducted by software with different values of all three variables. The response (Y) was taken as percentage reduction of Cr(VI). The ANOVA table was obtained by inserting responses into the software.

### 2.7. Solar Photocatalytic Treatment of Real Wastewater

Under the optimized reaction conditions, the real wastewater containing Cr(VI) was treated with the ZnO/CuO/PE-based PMR. The working volume was 50 mL and solar flux was 5–6 kW h/m^2^. The extent of reduction was measured by UV/Vis spectroscopy (CECIL CE 7200, Germany) and the complexation of Cr(VI) and Cr(III) before and after solar photocatalytic reduction.

## 3. Result and Discussion

### 3.1. Characterization of the ZnO/CuO/PF-Based PMR

The results of the structural morphological, optical, and thermal characterization of ZnO/CuO collected from the surface of the PMR and ZnO/CuO/PFPMR are discussed below.

#### 3.1.1. Morphological Characterization of the ZnO/CuO/PF-Based PMR

**Figure 2:**
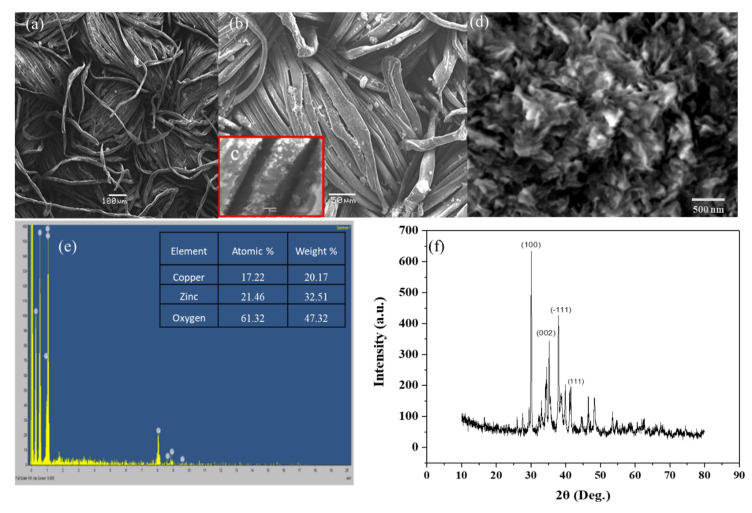
Structural and morphological characterization of ZnO/CuO nanoflakes grafted onto the surface of strands of PF at (**a**) 1KX, (**b**) 5 KX, and (**c**) 10 KX. (**d**) Nanoflakes of ZnO/CuO composite. (**e**) EDX spectrum and (**f**) diffractogram of ZnO/CuO composite grafted on polyester fabric.

The polyester fabric grafted with ZnO/CuO composite was observed to be densely covered with flake-like nanostructures (Figure 2a,b). The average dimensions of the structures calculated by ImageJ software were 67 × 56 × 12 nm^3^. The magnified micrograph of a few strands of fabric showed nanoflakes grafted densely onto the strands of polyester fabric (Figure 2c). The even and dense covering of the functionalized polyester fabric can be attributed to an alkali treatment as pores had appeared on the surface of the fabric due to etching. The alkali treatment of the fabric resulted in surface roughness, which might have caused the generation of sites for the easy penetration of nanoparticles. The pointed tapering ends of the thin flakes consist of ZnO/CuO composite.

Morphological and surface analyses of ZnO/CuO/PF nanocomposite were studied through SEM images. Flake-like monomorphous nanostructures were obtained (Figure 2d), showing a geometry suitable for photocatalytic applications as it provides a high surface area with a large number of active sites, offering channels for electron movement during photocatalytic reactions [33]. The elemental analysis and purity of ZnO/CuO/PF nanocomposite were studied through the EDX spectrum, giving peaks of Zn, Cu, and O with Zn and Cu intensities in the 1:1 ratio as both participate equally in the synthesis of the composite; a greater oxygen intensity was also revealed in ZnO/CuO/PF composite fabrication. The EDX results confirmed the authenticity of the composite formation and the high purity of the nanocomposite shown in Figure 1e. According to atomic percentage and weight percentage data obtained from EDX, almost the same concentrations of Cu^+2^ and Zn^+2^ were present owing to their similar cationic sizes. The higher concentration of oxygen than the two component cations revealed that an oxide of both zinc and copper was formed as a composite. Moreover, a high concentration of surface adhered oxygen as hydroxyl groups was indicated.

#### 3.1.2. Structural Characterization of ZnO/CuO Grafted on PMR

The crystal structure of ZnO/CuO nanocomposite was studied through XRD and the observed diffraction design was consistent with the information accessible in JCPDS cards (JCPDS; 36-1451 for ZnO, JCPDS; 05-0661 for CuO), demonstrating the fabricated sample as crystalline, and the phase purity and phase separation between ZnO and CuO is clearly visible (Figure 2f), proving the presence of both ZnO and CuO. The diffractogram shows peaks at the 33.96° (002) plane, 31.31° (100) plane, and 35.79° (101) plane, revealing the presence of ZnO and peaks at the 38.29° (111) and 35.05° (111) plane are due to CuO, proving its consistency with the standards values [34]. The average crystallite size of ZnO/CuO/PF nanocomposite was calculated numerically as (L) by using the Debye–Scherrer formula as given in the following equation [35,36].

(3) $$L=\frac{k\lambda }{\beta \cos\theta }$$

Taking the value of β = FWHM from the graph, the calculated crystallite size of ZnO/CuO/PE was found to be 13.5 nm.

#### 3.1.3. Optical Properties of ZnO/CuO Grafted onto the PMR

**Figure 3:**
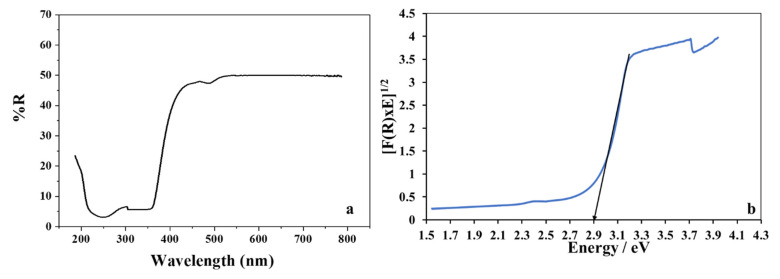
(**a**) Diffuse reflectance spectrum for % reflectance of ZnO/CuO and (**b**) band gap energy for ZnO/CuO.

The diffused reflectance spectrum indicated that almost 50% of the sunlight was reflected, confirming the high absorption of solar radiation by the composite of the fabricated material (Figure 3a). The bandgap edge was observed to be at 365 nm according to the DRS spectrum of ZnO/CuO composite. Intrinsic ZnO, having a band gap at 3.2 eV, cannot absorb visible radiation [37,38]. On the other hand, ZnO/CuO/PF exhibited an enhanced light harvesting capability in the solar spectrum. The band gap energy of ZnO/CuO was calculated by a Kubelka–Munk plot using the relation given below [39].

[F(*R*∞)]^1/2^ = *A* (*hv* − *Eg*) (4)

where the absorption coefficient (*A*) is related through Taucs relation, *R*∞ is the diffused reflectance, (*R*∞) is the Kubelka–Munk function, and *Eg* represents the band gap energy. Using this Kubelka–Munk relation, the band gap energy for ZnO/CuO/PF was found to be 2.9 eV (Figure 3b). The decrease in the bandgap can be attributed to the induction of inter-band energy states below the conduction band (CB). Consequently, an electron as a charge carrier requires less energy to become excited from the valence band (VB) compared to the CB.

#### 3.1.4. Thermal Stability of ZnO/CuO Grafted onto the PMR

**Figure 4:**
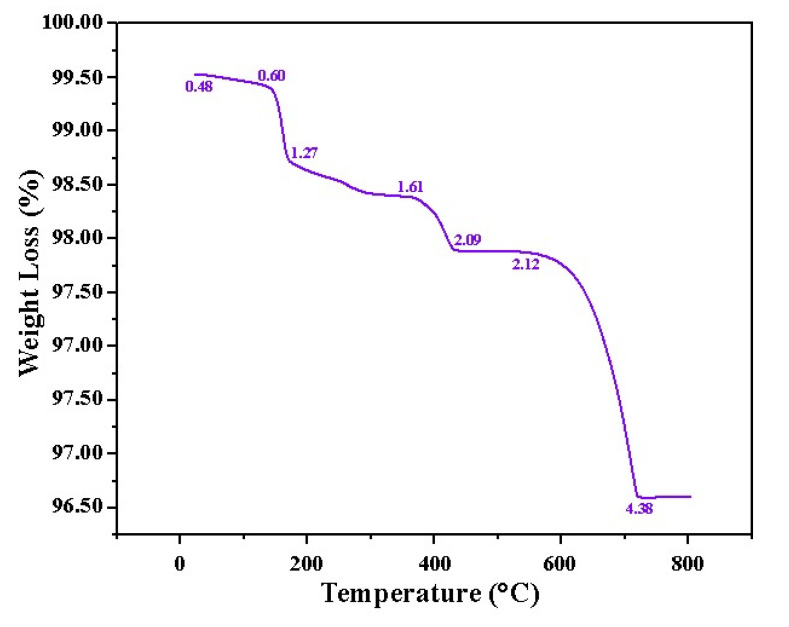
TGA curve of ZnO/CuO indicating the high thermal stability of nanocomposite.

The thermal stability of ZnO/CuO nanocomposite was measured by TGA and the curve obtained indicated that the initial weight loss for the nanocomposite was observed between 100–200 °C due to the evaporation of adsorbed water molecules from the surface of the composite (Figure 4). Moreover, dehydration of Cu(OH)_2_ to CuO was found to occur between 190 and 210 °C. The second gradual weight loss of 1.27–1.61% took place from 200–410 °C, then a greater weight loss of 2.00% appeared in the temperature range of 410–480 °C due to the evaporation of chemically bound water, indicating the decomposition of Zn(OH)_2_. The third and most prominent weight loss was observed at 610–700 °C (weight loss of 4.38%) due to the complete loss of water from the sample. The stability in the overall weight loss up to 800 °C can be deduced from only a 4.38% total weight loss. The thermal stability is attributed to the efficient catalytic activity of the composite grafted on the ZnO/CuO/PF PMR. Previous studies have reported similar results [40].

### 3.2. Characterization of the ZnO/CuO/PF-Based PMR for Surface Functionalization

**Figure 5:**
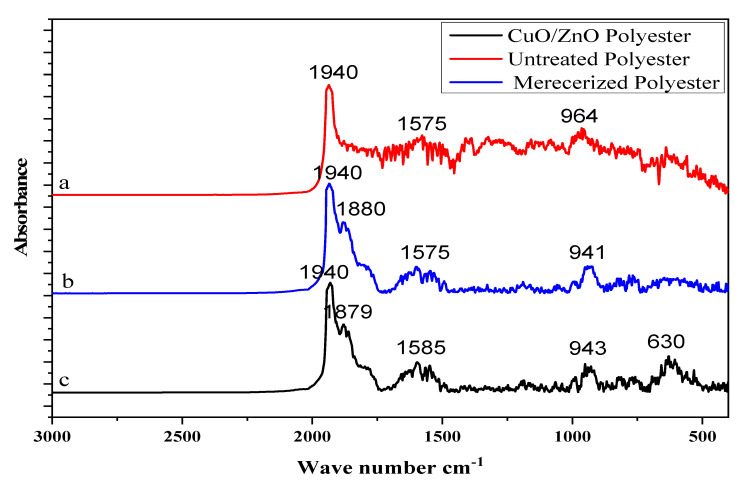
FTIR spectra of (**a**) untreated polyester, (**b**) mercerized polyester, and (**c**) ZnO/CuO/PF PMR.

The untreated polyester fabric, mercerized polyester, and ZnO/CuO/PF-based PMR were subjected to FTIR analysis. According to the spectra obtained, the band observed at 1940 cm^−1^ appeared due to carbonyl groups. The bands at 1575 cm^−1^ (Figure 5) correspond to C = N and C–N groups present in benzenoid and quinoid structures and OH bending in the COOH group. Peaks at 964, 941, and 943 cm^−1^ exhibited in all spectra are due to the vinyl (C–H) group. No peak appeared at 630 cm^−1^ for the untreated and mercerized polyester, but it did for ZnO/CuO grafted on mercerized polyester. This small and broad peak indicated the grafting of the nanocomposite onto the surface of mercerized polyester in a small amount, appearing due to stretching of the Zn–O and Cu–O bond. Similar results have been reported for polymers [41].

### 3.3. Solar Photocatalytic Reduction of Chromium by RSM

The response of the solar photocatalytic reduction of Cr(VI) was collected by executing the experimental runs provided by a complete polynomial quadratic model as the central composite design (CCD). Design Expert V.7.0.0 (Stat-Ease Inc., Minneapolis, MN, USA) was used to calculate the interactive effect of all three variables while using ZnO/CuO/PF as a PMR under natural sunlight. After the solar photocatalytic treatment, all 20 samples were collected and absorbance was measured by UV/Vis spectroscopy (Table 1) [42].

#### Optimization of Operational Parameters by Response Surface Methodology

While taking the photocatalyst load constant (58 micro g/cm^3^) as the solar photocatalytic membrane reactor and solar flux (5–6 kW h/m^2^), variable operational parameters, i.e., pH (5–9), initial Cr(VI)concentration (10–50 ppm), and solar irradiation time (2–6 h), were statistically optimized through response surface methodology (RSM), a statistical and mathematical tool for achieving the maximum reduction of Cr(VI). The basic equation for the selected quadratic model that represents the relationship between variables and their interactive effect on the percentage reduction of Cr(VI) is given in Equation (1). Furthermore, percentage reduction was calculated from absorbance to get the maximum Cr(VI) percentage reduction of standard solutions (Table 1).

The ANOVA table includes a lower value of Prob F > 0.005 as the F value of 20.67 with 0.01% chances of noise, ensuring the significant effect of variables on the response. Moreover, the value of *p* < 0.0001 with Pred. R^2^ = 0.7537 and Adj. R^2^ = 0.9031 agreement ensures that the model is greatly significant, and the best predictability was shown by the insignificant lack of the fit test (Table 2). The parameter optimization expression for response surface methodology is given in Equation (5).


(5) $$Y=+73.19+3.01A+4.49B+20.16-0.37\mathrm{AB}-4.12\mathrm{AC}-0.37\mathrm{BC}-0.87A^{2}+3.02B^{2}-8.65C^{2}$$

The above expression represents Y as percentage reduction and X1, X2, and X3 are the variables, i.e., oxidant concentration, pH, and irradiation time.

### 3.4. Interactive Effects of Operational Variables of Solar Photocatalytic Reaction

**Figure 6:**
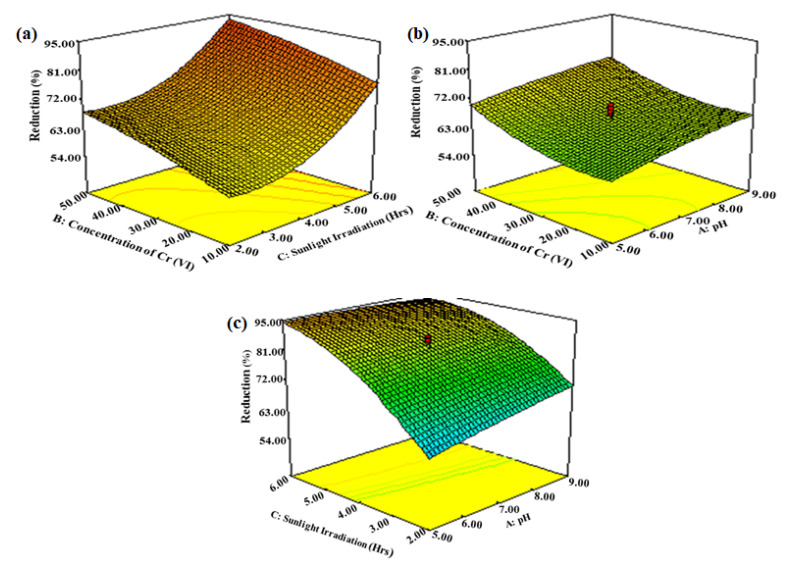
(**a**) RSM 3D interactive plot of irradiation time and initial concentration of Cr(VI), (**b**) interactive plot of pH and initial concentration of Cr(VI), and (**c**) interactive effect of irradiation time and pH.

Three-dimensional graphics represent the regression equation for optimizing reaction conditions. The 3D response surface interaction plots along with the interactive effect of dependent variables’ interactions are represented below (Figure 6).

#### 3.4.1. Interactive Effect of Initial Concentration of Cr(VI) and Irradiation Time

The initial concentration of Cr(VI) and solar irradiation time showed an interactive effect, proving the linear relationship between dependent variables in the case of percentage reduction of Cr(VI) as shown in Figure 6a. An increase in the initial concentration offered a higher percentage reduction up to 50 ppm of concentration due to the rapid coverage of available active sites by ions and the enhanced rate of photocatalysis. Furthermore, the photocatalyst surface initially had a larger amount of active sites for Cr(VI) adsorption followed by reduction, but the reduction efficiency decreased slightly after some time of increasing metal ion concentration adsorbed on vacant sites; they all became occupied in 2–4 h according to the 3D surface plot [43].

#### 3.4.2. Interactive Effect of Initial Concentration of Cr(VI) and pH

The initial concentration of Cr(VI) and pH interactive effect has exhibited an indirect relationship in the case of the percentage reduction of Cr(VI) as represented in Figure 6b. The estimated Cr(VI) reduction demonstrated a lesser reduction with an increasing concentration of Cr(VI), as exceeding the optimum Cr(VI) concentration prohibited the approach of more Cr(VI) ions to the active sites of the photocatalyst. Another important variable is pH, which affects the reduction rate of Cr(VI) by controlling the surface charge of the photocatalyst during the photocatalytic reaction. The rate of photocatalysis decreased with an increase in pH. Furthermore, Cr(VI) to Cr(III) reduction generated hydroxyls in the alkaline medium and utilized protons. It can be observed that in the acidic medium, the best results were obtained in the pH range of 5–7 [44].

(6) $${\mathrm{Cr}}_{2}O_{7}^{2-}+14H^{+}+6e^{-}\to 2\mathrm{Cr}3^{+}+7H_{2}O$$

(7) $${\mathrm{Cr}}_{2}O_{7}^{2-}+14H_{2}O+3e^{–}\to \mathrm{Cr}(\mathrm{OH}{)}_{3}+5\mathrm{OH}$$

#### 3.4.3. Interactive Effect of pH and Irradiation Time

The pH and irradiation time interactive effect on Cr(VI) percentage reduction is represented in Figure 6c, depicting a linear relationship between these independent variables. In alkaline medium, the surface of ZnO/CuO holds additional negative charges, since it is electrostatically repelled by the negatively charged species, such as Cr_2_O_4_^2−^, HCrO_4_^−^, CrO_4_^2−^, and neutral H_2_CrO_4_ species, showing a decreased extent of Cr(VI) adsorption on the ZnO/CuO/PE surface at a higher pH range, whereas a positively charged photocatalyst at neutral pH acts as a Lewis acid, thus promoting the rate of photocatalytic reduction of Cr (VI) to Cr (III), also confirmed from the 3D RSM plot. The reduction efficiency of Cr(VI) was enhanced by a higher irradiation time, but after a certain limit, all active sites were blocked, decreasing the reduction [45]. Since the best results were obtained at pH 5–7 and a sunlight irradiation time of 6 h, it can be concluded that in a slightly acidic medium, up to 95% reduction of Cr(VI) can be obtained.

### 3.5. Complexation of Cr(VI) and Cr(III) Complexes

**Figure 7:**
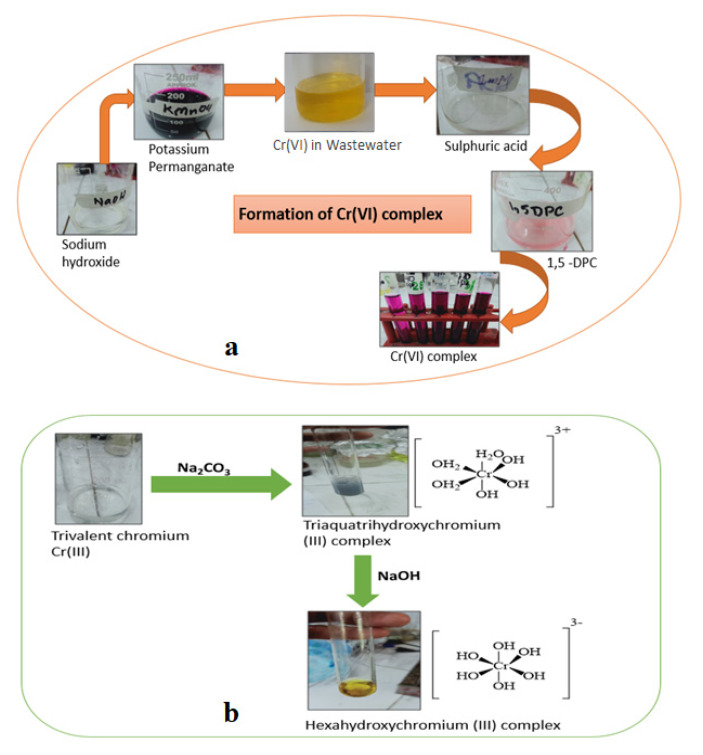
(**a**) Formation of Cr(VI) complex and (**b**) formation of Cr(III) complexes.

The standard solution of Cr(VI) in the concentration range of 10–50 ppm and wastewater were subjected to complexation, and the variation in intensity of the reddish violet color indicated the differences in concentration of Cr(VI). Similarly, Cr(VI) in wastewater was also subjected to a complexation reaction. The steps given in Figure 7a show the formation of a hexavalent chromium complex with 1,5-diphenylcarbazide. After executing a solar photocatalytic reduction reaction using the ZnO/CuO/PF PMR, Cr(VI) was reduced to the trivalent chromium, which was further reacted with complexing reagents as given in Figure 7b. The end product was a hexahydroxy chromium III complex of greenish yellow color which reduced the concentration of Cr(III) from standard solutions of Cr(VI) and treated wastewater.

**Figure 8:**
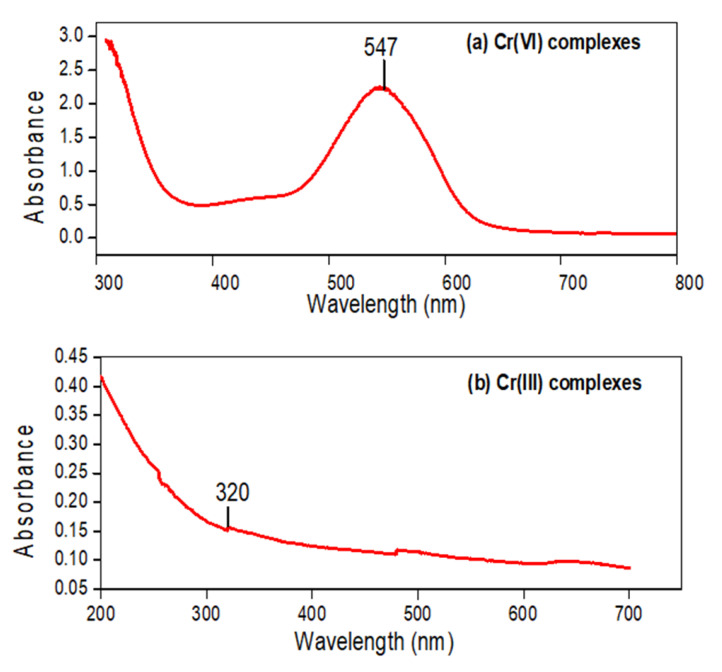
UV/Vis spectra of (**a**) Cr(VI) and (**b**) Cr(III) complexes for untreated and treated wastewater.

UV/Vis spectroscopy analysis was used for the determination of hexavalent and trivalent chromium complexes. Cr(VI) formed a red-violet color complex with 1,5-diphenylcarbazide, showing a maximum absorption at 547 nm wavelength [46,47,48,49]. The high peak of the Cr(VI) complex delineated the large concentration of Cr(VI) in the wastewater sample. After the solar photocatalytic reduction reaction, no reddish violet color appeared upon complexation, indicating an almost complete conversion of Cr(VI) to Cr(III). On executing the complexation reaction of Cr(III) in treated wastewater, trivalent chromium formed a octahedral complex of a greenish yellow color and showed the maximum absorbance at 320 nm. The very low peak of Cr(III) indicated the reduced concentration of trivalent chromium in the treated wastewater [50]. Figure 8a,b shows the UV/Vis spectra of the Cr(VI) and Cr(III) complexes, respectively.

The photocatalytic reduction of Cr (VI) to Cr (III) was confirmed by the formation of complexes of chromium. Hexavalent chromium exists in chromate and dichromate forms. The potassium dichromate (orange color) was first reduced to Cr^3+^. Then, after the addition of Na_2_CO_3_ and NaOH, a greenish yellow colored hexa-aqua chromium(III) ion, a complex formed by Cr^3+^ in aqueous solution, was obtained [51]. Thus, it is clear from the results that Cr^3+^ forms Cr(H_2_O)^3+^_6_, {Cr(H_2_O)_3_(OH)_3_}, and {Cr(OH)_6_}^3−^ octahedral complexes in the aqueous solution [52,53].

### 3.6. Photocatalytic Reduction of Cr(VI) to Cr(III) in Real Wastewater

**Figure 9:**
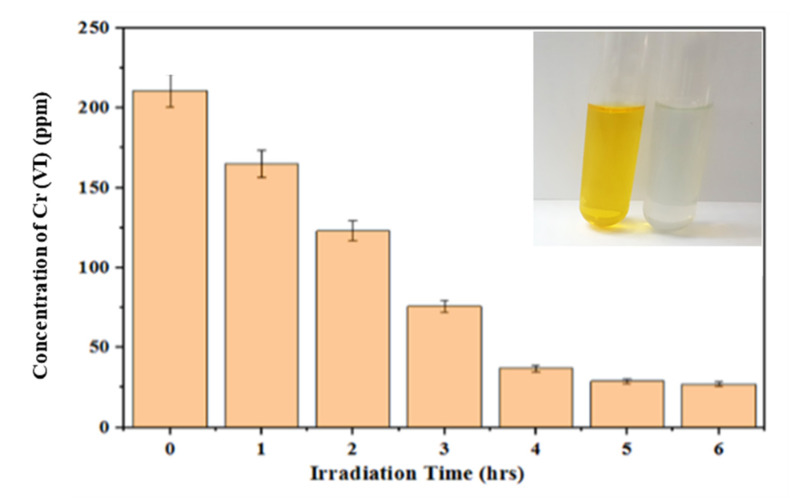
Decrease in concentration of Cr(VI) after solar photocatalytic reduction reaction.

The concentration of Cr(VI) in the real wastewater sample was measured by UV/Vis spectroscopy to be 200 ppm, taking the average. Under the optimized conditions, a solar photocatalytic reaction was carried out using the ZnO/CuO/PF PMR. The real wastewater was yellow in color due to Cr(VI); after the photocatalytic reduction reaction under natural sunlight, the color of the wastewater changed, indicating the decrease in the concentration of Cr(VI). The continuous exposure of the real wastewater to solar flux (5–6 kW h/m^2^) for six hours (10 a.m. to 4 p.m.) with a photocatalyst load (58 ± 2 μg/cm^3^) as the solar photocatalytic membrane reactor showed an impressive decrease in Cr(VI), which was observed to be 24 ppm. The large decrease in the concentration of Cr(VI) rendered the water reusable for irrigation and industrial processes (Figure 9)**.**

The concentration of chromium in real wastewater as percentage removal was estimated by AAS for the decrease in concentration of Cr(VI) after the solar photocatalytic reduction reaction. The concentration of Cr as Cr(VI) and Cr(III) decreased with respect to time. The samples collected from the reactor mixture exposed to natural sunlight, after regular intervals, were aspirated in AAS for determination of the Cr concentration (Table 3). It is obvious from the results obtained that Cr concentration declined sharply after 3 h and become almost constant after 5 h. The increase in the concentration of H^+^ on proceeding the reaction caused an increase in the reduction of Cr(VI). Furthermore, Cr(III) as a product of the solar photocatalytic reaction adsorbed onto the surface of ZnO/CuO/PF, decreasing the overall concentration of Cr in the wastewater. The maximum decrease in percentage removal of Cr was observed to be 25 ppm.

### 3.7. Reusability of ZnO/CuO/PF

**Figure 10:**
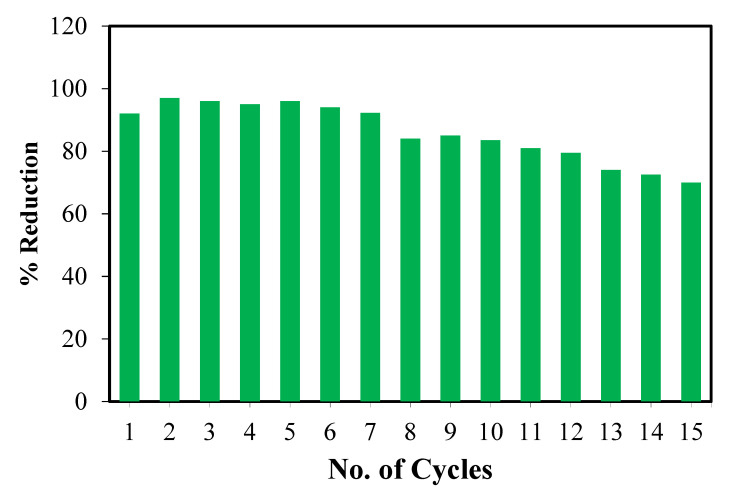
Reusability of ZnO/CuO/PF for reduction of Cr (VI) to Cr (III).

The basic purpose of the immobilization of the photocatalyst (ZnO/CuO) onto the polyester substrate is to make it easily reusable and cost-effective. Reusability of ZnO/CuO/PF was determined as shown in Figure 8. The efficiency of ZnO/CuO/PF was retained up to seven cycles and then a gradual decrease in the performance of the photocatalyst occurred (Figure 10). Other researchers have reported that, in the case of using PMR consisting of pure ZnO, only an 81% reduction of Cr (VI) to Cr (III) could be obtained [54]. Moreover, even on doping of ZnO with tin (Sn/ZnO), no measurable increase (80% removal) in the photocatalytic activity of the material was observed [55]. Conclusively, in comparison to the results of other research, it is obvious that ZnO/CuO/PF is a more effective PMR, as a 95% reduction of Cr(VI) was obtained, which decreased to 78% after 15 cycles.

## 4. Conclusions

ZnO/CuO/PF was successfully designed by the SILAR method, as ZnO/CuO nanocomposite was grafted onto polyester fabric as a highly durable and chemical resistant photocatalytic membrane reactor. The surface binding forces of polyester with the nanocomposite were enhanced by mercerization using 40 g/L of NaOH. The fabricated PMR is a novel material to enhance the photocatalytic reduction application, suitable for the reuse of wastewater after reduction of Cr (VI) to Cr (III). The characterization of synthesized CuO/ZnO nanocomposite proved its high purity and crystallinity, whereas its flake-like morphology and rough texture show a great potential for photocatalytic applications. The optical properties of CuO/ZnO nanocomposite indicated the high harvesting power of solar radiation due to the optimized low band gap energy of 2.9 eV. The thermal stability of ZnO/CuO was also determined to be high, with only a 4.38% weight loss. The surface characterization of ZnO/CuO/PF PMR was executed to confirm the grafting of CuO/ZnO nanocomposite onto the surface of functionalized polyester fabric. The extent of Cr(VI) reduction was maximum at pH 6, when the initial concentration of Cr(VI) was 30 ppm and the solution was irradiated for 4 h in natural sunlight. The real wastewater was treated under optimized conditions, and up to a 97% reduction in the concentration of Cr(VI) was observed. The extent of the reduction of the concentration of Cr(VI) to Cr(III) was evaluated by UV/Vis spectrophotometry. Complexation of Cr(VI) and Cr(III) with 1,5-diphenylcarbazide and their estimation of concentration at 547 nm at 320 nm, respectively, confirmed the reduction of Cr(VI) to Cr(III). A further decrease in the concentration of Cr(III) in the treated wastewater was observed due to its complexation in alkaline medium. The percentage removal of Cr attained in real wastewater as determined by AAS after the regular time interval was 25% after 6 h. The reusability of the PMR was determined by repeating the photocatalytic reduction of 15 batches of wastewater containing Cr (VI), with a slight decrease in efficiency after 8 cycles, and a 78% reduction was obtained for the 15th cycle. This cost-effective and reusable PMR can be considered as an efficient material to convert Cr(VI) into innocuous Cr(III) to render wastewater reusable for irrigation and industrial processes.

## Figures and Tables

**Table 1:** Experimental results of solar photocatalytic reduction of Cr(VI) for runs provided by central composite design.

Run	pHbol	Initial Concentration of Cr(VI) (ppm)	Sunlight Irradiation Time (h)	Reduction (%)
1	9.00	10	6.00	87
2	9.00	50	6.00	84
3	5.00	10	2.00	38
4	3.64	30	4.00	71
5	7.00	30	4.00	77
6	7.00	30	4.00	74
7	7.00	30	4.00	97
8	7.00	30	4.00	76
9	5.00	50	2.00	32
10	7.00	30	4.00	75
11	10.36	30	4.00	77
12	7.00	63.5	7.36	83
13	9.00	50	2.00	41
14	7.00	30	4.00	61
15	9.00	10	2.00	54
16	5.00	50	6.00	83
17	7.00	3.5	4.00	75
18	7.00	30	4.00	73
19	5.00	10	6.00	89
20	7.00	30	0.64	21

**Table 2:** ANOVA for response surface quadratic model.

Source	Sum of Squares	df	Mean Square	F Value	*p*-Value Prob > F	
Model	7384.73	9	820.53	20.67	<0.0001	Significant
A—Initial concentration of Cr(VI)	275.72	1	275.72	6.94	0.0249	
B—pH	123.63	1	123.63	3.11	0.1081	
C—irradiation time	5548.44	1	5548.44	139.75	<0.0001	
AB	1.13	1	1.13	0.028	0.8697	
AC	136.12	1	136.12	3.43	0.0938	
BC	1.13	1	1.13	0.028	0.8697	
A^2^	10.90	1	10.90	0.27	0.6117	
B^2^	131.39	1	131.39	3.31	0.0989	
C^2^	1077.75	1	1077.75	27.15	0.0004	
Residual	397.02	10	39.70			
Lack of fit	219.02	5	43.80	1.23	0.4128	Not significant
Pure error	178.00	5	35.60			
Cor total	7781.75	19				

**Table 3:** Concentration of Cr in ppm, determined by AAS with respect to time of solar photocatalytic reaction.

**Sample** **collection time (min)**	0	60	120	180	240	300
**Conc. of Cr (ppm)**	216	164	124	72	47	25

## Data Availability

The data will be made available upon request.

## References

[1] Baruah S., Pal K.S., Dutta J. (2012). Nanostructured zinc oxide for water treatment. Nanosci. Nanotechnol. Asia.

[2] Miranda-García N., Suárez S., Sánchez B., Coronado J., Malato S., Maldonado M.I. (2011). Photocatalytic degradation of emerging contaminants in municipal wastewater treatment plant effluents using immobilized TiO_2_ in a solar pilot plant. Appl. Catal. B Environ..

[3] Nagajyoti P., Lee K., Sreekanth T. (2010). Heavy metals, occurrence and toxicity for plants: A review. Environ. Chem. Lett..

[4] Radhakrishnan A., Rejani P., Khan J.S., Beena B. (2016). Effect of annealing on the spectral and optical characteristics of nano ZnO: Evaluation of adsorption of toxic metal ions from industrial waste water. Ecotoxicol. Environ. Saf..

[5] Ali S., Noureen S., Shakoor M.B., Haroon M.Y., Rizwan M., Jilani A., Arif M.S., Khalil U. (2020). Comparative evaluation of wheat straw and press mud biochars for Cr(VI) elimination from contaminated aqueous solution. Environ. Technol. Innov..

[6] Rai V., Vajpayee P., Singh S.N., Mehrotra S. (2004). Effect of chromium accumulation on photosynthetic pigments, oxidative stress defense system, nitrate reduction, proline level and eugenol content of *Ocimum tenuiflorum* L.. Plant Sci..

[7] Ghani A. (2011). Effect of chromium toxicity on growth, chlorophyll and some mineral nutrients of *Brassica juncea* L.. Egypt. Acad. J. Biol. Sci..

[8] Sezgin N., Yalçın A., Köseoğlu Y. (2016). MnFe_2_O_4_ nano spinels as potential sorbent for adsorption of chromium from industrial wastewater. Desalination Water Treat..

[9] Zakria H.S., Othman M.H.D., Kamaludin R., Sheikh Abdul Kadir S.H., Kurniawan T.A., Jilani A. (2021). Immobilization techniques of a photocatalyst into and onto a polymer membrane for photocatalytic activity. RSC Adv..

[10] Zakria H.S., Othman M.H.D., Kamaludin R., Jilani A. (2021). Study on the effect of air gap on physico-chemical and performance of PVDF hollow fibre membrane. IOP Conf. Ser. Mater. Sci. Eng..

[11] Tan Y., Chen M., Hao Y. (2012). High efficient removal of Pb (II) by amino-functionalized Fe_3_O_4_ magnetic nano-particles. Chem. Eng. J..

[12] Gehrke I., Geiser A., Somborn-Schulz A. (2015). Innovations in nanotechnology for water treatment. Nanotechnol. Sci. Appl..

[13] Mohsin M., Bhatti I.A., Ashar A., Khan M.W., Farooq M.U., Khan H., Hussain M.T., Loomba S., Mohiuddin M., Zavabeti A. (2021). Iron-doped zinc oxide for photocatalyzed degradation of humic acid from municipal wastewater. Appl. Mater. Today.

[14] Inderyas A., Bhatti I., Ashar A., Ashraf M., Ghani A., Yousaf M., Mohsin M., Ahmad M., Rafique S., Masood N. (2020). Synthesis of immobilized ZnO over polyurethane and photocatalytic activity evaluation for the degradation of azo dye under UV and solar light irardiation. Mater. Res. Express.

[15] Ashar A., Iqbal M., Bhatti I.A., Ahmad M.Z., Qureshi K., Nisar J., Bukhari I.H. (2016). Synthesis, characterization and photocatalytic activity of ZnO flower and pseudo-sphere: Nonylphenol ethoxylate degradation under UV and solar irradiation. J. Alloys Compd..

[16] Shaheen M., Bhatti I.A., Ashar A., Mohsin M., Nisar J., Almoneef M.M., Iqbal M. (2021). Synthesis of Cu-doped MgO and its enhanced photocatalytic activity for the solar-driven degradation of disperse red F3BS with condition optimization. Z. Für Phys. Chem..

[17] Sherly E., Vijaya J.J., Kennedy L.J. (2015). Visible-light-induced photocatalytic performances of ZnO–CuO nanocomposites for degradation of 2, 4-dichlorophenol. Chin. J. Catal..

[18] Ishaq T., Yousaf M., Bhatti I.A., Ahmad M., Ikram M., Khan M.U., Qayyum A. (2020). Photo-assisted splitting of water into hydrogen using visible-light activated silver doped g-C3N4 & CNTs hybrids. Int. J. Hydrogen Energy.

[19] Jilani A., Othman M.H.D., Ansari M.O., Oves M., Hussain S.Z., Khan I.U., Abdel-wahab M.S. (2019). Structural and optical characteristics, and bacterial decolonization studies on non-reactive RF sputtered Cu–ZnO@ graphene based nanoparticles thin films. J. Mater. Sci..

[20] Mozia S., Tomaszewska M., Morawski A.W. (2007). Photocatalytic membrane reactor (PMR) coupling photocatalysis and membrane distillation—Effectiveness of removal of three azo dyes from water. Catal. Today.

[21] Parveen S., Bhatti I., Ashar A., Javed T., Mohsin M., Hussain M., Khan M., Naz S., Iqbal M. (2020). Synthesis, characterization and photocatalytic performance of iron molybdate (Fe_2_(MoO_4_)_3_) for the degradation of endosulfan pesticide. Mater. Express.

[22] Leong S., Razmjou A., Wang K., Hapgood K., Zhang X., Wang H. (2014). TiO_2_ based photocatalytic membranes: A review. J. Membr. Sci..

[23] Byung-Wan J., Park S.-K., Cheol-Hwan K. (2006). Mechanical properties of polyester polymer concrete using recycled polyethylene terephthalate. ACI Struct. J..

[24] Dhanasekaran V., Mahalingam T. (2012). Physical properties evaluation of various substrates coated cupric oxide thin films by dip method. J. Alloys Compd..

[25] Pathan H., Lokhande C. (2004). Deposition of metal chalcogenide thin films by successive ionic layer adsorption and reaction (SILAR) method. Bull. Mater. Sci..

[26] Rousselle M., Nelson M., Hassenboehler C., Legendre D. (1976). Liquid-ammonia and caustic mercerization of cotton fibers: Changes in fine structure and mechanical properties. Text. Res. J..

[27] Jiménez-García F., Londoño-Calderón C., Espinosa-Arbeláez D., Del Real A., Rodríguez-García M. (2014). Influence of substrate on structural, morphological and optical properties of ZnO films grown by SILAR method. Bull. Mater. Sci..

[28] Jilani A., Rehman G.U., Ansari M.O., Othman M.H.D., Hussain S.Z., Dustgeer M.R., Darwesh R. (2020). Sulfonated polyaniline-encapsulated graphene@graphitic carbon nitride nanocomposites for significantly enhanced photocatalytic degradation of phenol: A mechanistic study. New J. Chem..

[29] Zewdu F., Amare M. (2018). Determination of the level of hexavalent, trivalent, and total chromium in the discharged effluent of Bahir Dar tannery using ICP-OES and UV–Visible spectrometry. Cogent Chem..

[30] Lace A., Ryan D., Bowkett M., Cleary J. (2019). Chromium monitoring in water by colorimetry using optimised 1, 5-diphenylcarbazide method. Int. J. Environ. Res. Public Health.

[31] Chakraborty A., Mishra R. (1992). Speciation and determination of chromium in waters+. Chem. Speciat. Bioavailab..

[32] Hamada Y.Z., Carlson B.L., Shank J.T. (2003). Potentiometric and UV–Vis spectroscopy studies of citrate with the Hexaquo Fe^3+^ and Cr^3+^ metal ions. Synth. React. Inorg. Met. Org. Chem..

[33] Zaman S. (2012). Synthesis of ZnO, CuO and Their Composite Nanostructures for Optoelectronics, Sensing and Catalytic Applications.

[34] Haque Z., Ranjan P. (2014). Synthesis of ZnO/CuO nanocomposite and optical study of ammonia (NH_3_) gas sensing. Int. J. Sci. Eng. Res..

[35] Dustgeer M.R., Asma S.T., Jilani A., Raza K., Hussain S.Z., Shakoor M.B., Iqbal J., Abdel-wahab M.S., Darwesh R. (2021). Synthesis and characterization of a novel single-phase sputtered Cu_2_O thin films: Structural, antibacterial activity and photocatalytic degradation of methylene blue. Inorg. Chem. Commun..

[36] Madhusudhana N., Yogendra K., Mahadevan K.M. (2012). Photocatalytic degradation of violet GL2B azo dye by using calcium aluminate nanoparticle in presence of solar light. Res. J. Chem. Sci. ISSN.

[37] Mohsin M., Bhatti I.A., Ashar A., Mahmood A., ul Hassan Q., Iqbal M. (2020). Fe/ZnO@ ceramic fabrication for the enhanced photocatalytic performance under solar light irradiation for dye degradation. J. Mater. Res. Technol..

[38] Ashar A., Bhatti I.A., Ashraf M., Tahir A.A., Aziz H., Yousuf M., Ahmad M., Mohsin M., Bhutta Z.A. (2020). Fe^3+^@ ZnO/polyester based solar photocatalytic membrane reactor for abatement of RB5 dye. J. Clean. Prod..

[39] Jilani A., Hussain S.Z., Ansari M.O., Kumar R., Dustgeer M.R., Othman M.H.D., Barakat M.A., Melaibari A.A. (2021). Facile synthesis of silver decorated reduced graphene oxide@zinc oxide as ternary nanocomposite: An efficient photocatalyst for the enhanced degradation of organic dye under UV–visible light. J. Mater. Sci..

[40] Mohammadi-Aloucheh R., Habibi-Yangjeh A., Bayrami A., Latifi-Navid S., Asadi A. (2018). Green synthesis of ZnO and ZnO/CuO nanocomposites in Mentha longifolia leaf extract: Characterization and their application as anti-bacterial agents. J. Mater. Sci. Mater. Electron..

[41] Ahmad M.A., Puad N.A.A., Bello O.S. (2014). Kinetic, equilibrium and thermodynamic studies of synthetic dye removal using pomegranate peel activated carbon prepared by microwave-induced KOH activation. Water Resour. Ind..

[42] Kamsonlian S., Shukla B. (2013). Optimization of process parameters using Response Surface Methodology (RSM): Removal of Cr (VI) from aqueous solution by wood apple shell activated carbon (WASAC) Res. J. Chem. Sci..

[43] Alswata A.A., Ahmad M.B., Al-Hada N.M., Kamari H.M., Hussein M.Z.B., Ibrahim N.A. (2017). Preparation of Zeolite/Zinc Oxide Nanocomposites for toxic metals removal from water. Results Phys..

[44] Assadi A., Dehghani M.H., Rastkari N., Nasseri S., Mahvi A.H. (2012). Photocatalytic reduction of hexavalent chromium in aqueous solutions with zinc oxide nanoparticles and hydrogen peroxide. Environ. Prot. Eng..

[45] Saien J., Azizi A., Soleymani A.R. (2014). Photocatalytic reduction of ni (II) ions using low amounts of titania nanoparticles: RSM modelling, kinetic. Iran. J. Toxicol..

[46] Wiryawan A., Retnowati R., Burhan P., Syekhfani S. (2018). Method of analysis for determination of the chromium (Cr) species in water samples by spectrophotometry with diphenylcarbazide. J. Environ. Eng. Sustain. Technol..

[47] Suryati L., Sulistyarti H., Atikah A. (2015). Development of spectrophotometric method for determination of chromium species using hypochlorite agent based on the formation of Cr (VI)-Diphenylcarbazide complex. J. Pure Appl. Chem. Res..

[48] Kocurek P., Vašková H., Kolomaznik K., Bařinová M. (2015). Hexavalent chromium determination in waste from leather industry using spectrophotometric methods. Wseas. Trans. Environ. Dev..

[49] Zhao X., Sui Z.H., Zhang J.B. (2010). Determination of Trace Chromium (VI) in Tanning Wastewater by Flow Injection Spectrophotometry. Adv. Mater. Res..

[50] Heena G., Rani S., Malik A., Kabir A. (2016). Speciation of Cr (III) and Cr (VI) Ions via Fabric Phase Sorptive Extraction for their Quantification via HPLC with UV Detection. J. Chromatogr. Sep. Tech..

[51] Lennartson A. (2014). The colours of chromium. Nat. Chem..

[52] Saha B., Orvig C. (2010). Biosorbents for hexavalent chromium elimination from industrial and municipal effluents. Coord. Chem. Rev..

[53] Theopold K.H. (2006). Chromium: Inorganic & Coordination Chemistry. Encycl. Inorg. Chem..

[54] Joshi K., Shrivastava V. (2011). Photocatalytic degradation of Chromium (VI) from wastewater using nanomaterials like TiO_2_, ZnO, and CdS. Appl. Nanosci..

[55] Hemalatha K., Manivel A., Kumar M.S., Mohan S.C. (2018). Synthesis and Characterization of Sn/ZnO Nanoparticles for Removal of Organic Dye and Heavy Metal. Biol. Chem..

